# The Preventive and Therapeutic Effects of Acute and Severe Inflammatory Disorders with Heparin and Heparinoid

**DOI:** 10.3390/biom14091078

**Published:** 2024-08-28

**Authors:** Ying Song, Yuxiang Wu, Fangfang Ding, Shuo Li, Yaojia Shen, Bingyan Yang, Xinran Tang, Lige Ren, Lirong Deng, Xuewen Jin, Yishu Yan

**Affiliations:** 1 School of Life Sciences and Health Engineering, Jiangnan University, Wuxi 214122, China6211504022@stu.jiangnan.edu.cn (Y.W.); 1162210109@stu.jiangnan.edu.cn (X.T.); 2 Medi-X Pingshan, Southern University of Science and Technology, Shenzhen 518118, China; 3 Shenzhen Hepalink Pharmaceutical Group Co., Ltd., Shenzhen 518057, China

**Keywords:** sepsis, heparin, COVID-19, anti-inflammation, structure and function relationship

## Abstract

Systematic inflammatory response syndrome (SIRS) and the accompanying sepsis pose a huge threat to human health worldwide. Heparin is a part of the standard supportive care for the disease. However, the molecular mechanism is not fully understood yet, and the potential signaling pathways that play key roles have not yet been elucidated. In this paper, the main findings regarding the molecular mechanisms associated with the beneficial effects of heparin, including inhibiting HMGB-1-driven inflammation reactions, histone-induced toxicity, thrombo-inflammatory response control and the new emerging mechanisms are concluded. To set up the link between the preclinical research and the clinical effects, the outcomes of the clinical trials are summarized. Then, the structure and function relationship of heparin is discussed. By providing an updated analysis of the above results, the paper highlights the feasibility of heparin as a possible alternative for sepsis prophylaxis and therapy.

## 1. Introduction

Systematic inflammatory response syndrome (SIRS) and the accompanying sepsis is not a disease but a life-threatening syndrome of systemic inflammatory response developed from local infections, trauma and acute organ injury [1,2]. About 50 million cases of sepsis occur globally each year with a mortality rate of approximately 20% [3]. 

**Figure 1:**
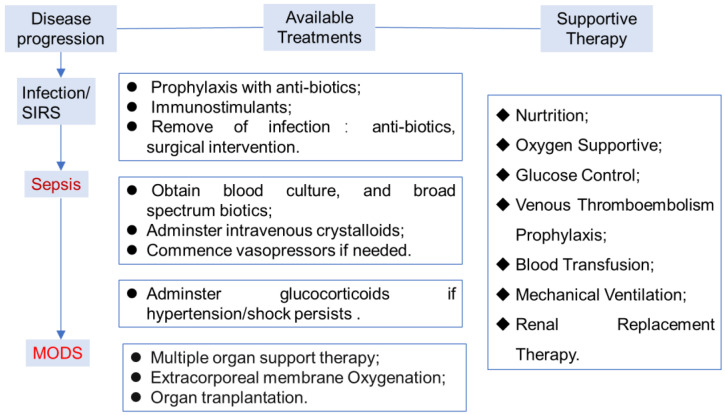
General procedures and approaches for sepsis management [10]. Multiple organ dysfunction syndrome, MODS.

Currently, several clinical intervention approaches including infection control, haemodynamic support and modulation of the host’s response have been employed for sepsis management (Figure 1) [4,5]. As infection is the underlying cause of sepsis, infection control with antibiotic therapy and surgical intervention to remove an infectious source is applicable to almost all patients. Broad-spectrum antibiotics are recommended to be administrated once sepsis is suspected to have happened, although this strategy might lead to the emergence of resistant organisms in recurrent infections.

Haemodynamic management is another basic approach for sepsis patients, which mainly includes fluid support due to the extensive occurrence of external and internal fluid loss. Vasoactive agents such as crystalloids and albumin are usually recommended in the presence of shock. A high dose of glucocorticoids shows significant mortality reduction, which is known to have various effects on reducing inflammation and enhancing immune response but is associated with worsening secondary infection and increased risk of death. Low-to-moderate doses of glucocorticoids do not improve survival or shock reversal in sepsis [6]. Noradrenaline would be used as a vasopressor agent, and dobutamine as an inotropic agent in case of myocardial depression. 

The host’s response to sepsis can be moderated using several approaches [5]. Extracorporeal blood purification systems were utilized to remove excess mediators and endotoxin; Vitamin C is recommended for its anti-inflammatory effects; renal replacement therapy would be used in the case of renal failure; ventilation strategy is required for adult patients with acute respiratory distress syndrome. 

Because of the complexity and heterogeneity of the pathology, it is unrealistic to expect a single drug or management strategy to cure the disease. For example, enteral nutrition is important in patients who have had abdominal sepsis, surgery or trauma. Stress ulcer prophylaxis with the use of histamine H2–receptor antagonists may decrease the risk of gastrointestinal hemorrhage [7]. Secondly, with the development of sepsis-specific biomarkers and molecular diagnostics for the assessment of the host’s response, precision-medicine and complex systems-medicine approaches related to immunotherapy of sepsis have advanced very rapidly [8]. However, none of the available strategies alone, or the integrated efforts have been demonstrated to reduce the mortality in the last 10 years, and clinical trials on large scales remain disappointing [9]. 

The pathophysiological mechanism of sepsis is related to the imbalance of inflammatory response and immune dysfunction, leading to a system-wide release of cytokines and pro-inflammatory mediators, which is tightly linked with the following highly coagulant state and multiple organ dysfunction. The therapeutic approaches that target the early events are vital for preventing inflammation exaggerations and the occurrence of sepsis [11,12]. Heparin is regarded as the most successful anticoagulant agent [13]. Meanwhile, accumulating evidence proves that the compound prevents SIRS and sepsis by exerting powerful anti-inflammation and immunomodulatory effects [14]. Especially, heparin was recommended as the first-line drug for prophylaxis against thromboembolism during the period of the COVID-19 crisis. The clinical outcome not only displays decreased levels of coagulant markers such as D-dimer and fibrinogen but also reduced cytokine releases such as IL-6 and TNF-α, thereby blocking a cytokine storm and improving the survival rate [15,16,17,18]. 

Despite the successful cases, several clinical studies were unable to demonstrate the beneficial effect of the 28-day mortality rate [19,20]. For instance, the results from back-to-back clinical trials of therapeutic anticoagulation with heparin in non-critically vs. critically ill COVID-19-infected patients are totally opposite. The therapeutic dose of heparin increases the probability of survival to hospital discharge with reduced use of cardiovascular or respiratory organ support as compared with usual-care thromboprophylaxis in non-critically ill patients [21]; whereas the same strategy leads to no effects of antithrombin and increased bleeding risks in critically ill patients [22]. 

The results on the one hand demonstrate that heparin represents an important advance in the prevention and therapy of critical illness; on the other hand, the clinical application of the drug is blind to some degree because the detailed mechanism of the anti-inflammatory effect is still not fully understood [23]. Recently, novel signaling pathways of clinical importance for heparin-inhibiting inflammation aggravation have been gradually discovered [24,25]. 

Moreover, heparin is a highly heterogeneous polysaccharide composed of various sulfated disaccharide repeating units of hexuronic acid and α-D-glucosamine with a molecular weight ranging from 3000 to 15,000 Da. The structural characteristics such as molecular weight distribution, number and position of the sulfate group determine the ability to bind with various proteins [26], which are in turn responsible for entirely different bioactivities [27]. The detailed structure–activity relationship is extensively discussed, but incompletely defined [28]. 

Herein, the possible molecular mechanisms involved in the therapeutic effects of heparin and heparinoid on severe acute inflammations will be analyzed, the results of recent clinical trials will be summarized, and the structure and function relationship will be discussed. By providing an updated analysis of the above results, this paper will explore the feasibility, suitability and efficiency of heparin as a possible alternative for sepsis prevention and therapy.

## 2. Recent Innovations of the Molecular Mechanism Research

A large number of studies have shown that the interaction between heparin and various biological targets depends on its specific structure. The anticoagulant activity of heparin, for instance, is closely related to a particular pentasaccharide structure, which specifically identifies Anti-thrombin III (AT-III) by electrostatic interaction. The binding event is followed by conformational change of AT-III and inhibition of F-Xa activation, thereby inhibiting the coagulation cascades [29]. With the ability to bind to multiple proteins, heparin was proven to moderate a wide range of inflammatory reactions, mainly through interaction with cell adhesion molecules (such as P-selectin and ICAM-1, etc.), which are determined as therapeutic targets during inflammation progression [30].

**Figure 2:**
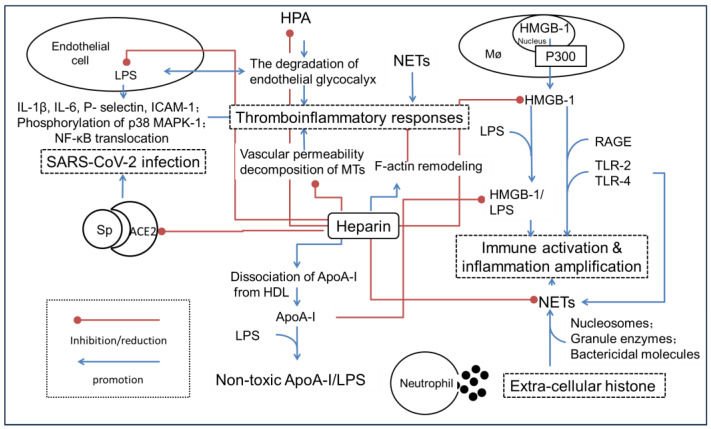
A simple schematic illustration of the possible molecular mechanisms that heparin alleviates the hyperinflammation state leading to SIRS or sepsis. Angiotensin-converting enzyme 2, ACE2; Apolipoprotein A-I, ApoA-I; intercellular cell adhesion molecule-1, ICAM-1; high-density lipoprotein, HDL; high mobility group protein-1, HMGB-1; haparanase, HPA; lipopolysaccharide, LPS; mitogen-activated protein kinase, MAPK; microtubule, MTs; neutrophil extracellular traps, NETs; advanced glycation end products, RAGE; severe acute respiratory syndrome coronavirus 2, SARS-CoV-2; surface-anchored trimer spike protein, Sp; Toll-like receptors-2/4, TLR-2/4.

As heparin has gradually become a main strategy for therapeutic mitigation of COVID-19-associated sepsis, the clinical evidence for inhibiting the inflammation cascade conspicuously increased. However, the anti-adhesion effects of heparin could not be directly attributed to the anti-excessive inflammation response. Several novel molecular mechanisms and possible drug targets have emerged apart from the previous discoveries (Figure 2).

### 2.1. Inhibition of HMGB-1-Driven Inflammation Reactions

HMGB-1 is a highly conserved DNA-binding factor expressed by all the nucleated eukaryotic cells. The protein was implicated in bending DNA to facilitate gene transcription, DNA replication and repair. When inflammation occurs, HMGB-1 is passively released from damaged cells or actively secreted from activated immune cells. The extracellular HMGB-1 was regarded as a member of damage-associated molecular patterns (DAMPs), a major mediator leading to inflammation amplification and disease deterioration [31]. Extracellular HMGB-1 interacts with RAGE after forming immuno-stimulatory complexes with endogenous or microbial co-factors. Moreover, HMGB-1 co-stimulates TLR-2/4 and activates the NF-κB signaling pathway, thus stimulating the rapid progress of infection until inflammatory storm occurrence [32,33].

Heparin alleviates inflammatory disorders by targeting the HMGB-1-associated signaling pathways, but the detailed mechanisms are still unknown. The first step for HMGB-1 secretion is transfer from the nucleus to the cytoplasm. The acetylation of HMGB-1 at the nuclear localization domains blocks its re-entry into the nucleus and determines the fate of secretion. Histone acetyltransferases, including p300, catalyze the lysine acetylation that is required for HMGB-1 secretion. Several groups have observed that heparin significantly inhibited HMGB-1 transfer from the nucleus to the cytoplasm [34]. Moreover, 2-O, 3-O desulfated heparin (ODSH) inhibits histone acetyltransferase p300 acetylation [35].

Secondly, heparin was found to interfere with the HMGB-1/RAGE axis by competitively binding HMGB-1 with extremely high affinity (KD = 4.5 × 10^−9^). Consequently, the conformation of HMGB-1 shows a marked change, and the affinity between HMGB-1 and the receptor RAGE decreases significantly. ODSH inhibits the binding of HMGB-1 to TLR-2/4 receptors, attenuating the detrimental effects of HMGB-1 on pulmonary host defense and reducing Pseudomonas aeruginosa-induced bacterial burden [35,36]. Moreover, heparin blocks the binding of HMGB-1 to the surface of macrophages and inhibits LPS-induced HMGB-1 amplified inflammatory responses through inhibiting phosphorylation of p38 and extracellular signal-regulated kinase-1/2 (ERK-1/2) [37]. 

Despite the fact that heparin mediates inflammatory conditions through HMGB-1 blockage, HMGB-1 alone is not the determinant factor leading to inflammation amplification; on the opposite, the complex of HMGB-1-LPS was considered a critical mediator of endotoxin lethality. Indeed, HMGB-1 directly binds to LPS, efficiently delivers LPS into the cytoplasm via RAGE, and inhibits Caspase-11 activation and protein gasdermin D cleavage, which augments the cellular injury and induces cellular death in severe acute pancreatic inflammation cases [38]. Whereas heparin derivatives inhibit the alarmin HMGB-1-LPS interaction, inhibiting the cytosolic delivery of LPS in macrophages and the subsequent Caspase-11-mediated lethality.

In light of these discoveries, the Jian Liu group synthesized an octadecasaccharide (18-mer heparan sulfate (HS) with a disaccharide repeating unit of -GlcNS-IdoA2S-) that protected mice from acute liver failure caused by paracetamol overdose. The protective effect is attributed to decreasing the complex HMGB-1-LPS formation and inhibiting the function of the HMGB-1/RAGE axis, resulting in neutrophil infiltration reduction in the liver. The findings show that 18-mer-HS possesses the same level of protective effect as the antibody against HMGB-1. It is noteworthy that the survival rate with 18-mer-HS treatment exceeds the HMGB-1 antibody group (53 vs. 90%) with a lethal dose of paracetamol [27,39].

### 2.2. Inhibition of Histone Toxicity

The histone family is usually located in the nucleus of eukaryotic cells and is divided into core histones (H2A, H2B, H3, H4) and adaptor histones (H1, H5). H1, H2A, and H2B are lysine-rich macromolecules, whereas H3 and H4 are arginine-rich macromolecules that constitute the basic structures of nucleosomes and chromatin. At physiological pH, histone has a high proportion of basic amino acid residues [40]. 

Normally, the concentration of extracellular histones is low. Histones would act as DAMPs after passive release by necrotic cells or via active release by neutrophils under pathological state. However, histones are extruded as the most abundant protein of NETs, a complex network consisting of nucleosomes, granule enzymes and bactericidal molecules [41]. The extra-cellular histone would signal through TLRs, myeloid differentiation primary response protein-88, NF-κB, and nucleotide-binding oligomerization domain-like receptor protein-3 inflammasomes to mediate a large amount of cytokine production and initiate organ damage and SIRS [42,43].

Heparin and its mimetic exhibit high affinity to histones. The binding of histones with heparin reduces their cytotoxicity in vitro, increasing the survival rate of mouse models of sterile inflammation induced by cecal ligation and puncture and LPS challenge [44]. Meanwhile, a low-anticoagulant fraction of heparin is able to rescue hepatic and renal dysfunction and prevent mortality of the animals challenged by continuous infusion of calf thymus histones [45]. As a heparinoid, chondroitin sulfate is also able to bind to extracellular histones and reduce the extracellular cell histone toxicity [46], although the effect substantially weakened compared with heparin, as the affinity remarkably reduces. As a result, heparinoids reduce the histone-induced inflammatory markers such as IL-6, IL-8, tissue factors and C3a. The ability to neutralize the cytotoxicity of histone is dependent on the molecular weight. Only the compounds with a Ms > 1.7 kDa neutralize histone-mediated toxicity for their high affinity to histone H3 [47]. 

### 2.3. Thrombo-Inflammatory Response Control

Although the inflammatory response and the activation of coagulation are two independent responses in a host’s defense against infection, the mechanisms truly work corporately in a synchronous manner [48]. Growing evidence proves that the coagulation dysfunction has a close relationship with excess inflammation occurrence. The so-called “thromboinflammatory responses” control was regarded as the initial task for sepsis disease therapy [49]. 

As a classical anti-coagulant agent, heparin and its mimetic display integrated efficacy targeting inflammation, coagulopathy and thrombocytopathy. For example, a concise assembly of heparin sodium with organosilicon quaternary ammonium surfactant to fabricate a multifunctional coating complex forms robust coatings to treat catheter-related bloodstream infections and thrombosis simultaneously. The coatings reduced thrombus adhesion by 60% in vitro and in vivo [50]. The key mechanism might involve the effects of endothelial cell glycocalyx integrity protection, NET inhibition and anti-coagulant activities. The associated targeting proteins include histone, HMGB-1 and HPA et al.

#### 2.3.1. Protect the Structure Integrity of the Endothelial Cells

The vascular endothelium is the first defense line for inflammation destruction, and a key regulator of thrombo-inflammation through expression of extensive coagulant and adhesive molecules. Heparin was shown to reduce vascular permeability in acute lung injury [51]. Impaired tight junction of the endothelium is a sign of sepsis-induced acute respiratory distress syndrome and acute lung injury. Heparin protects tight junctions associated proteins such as claudin-5, occludin, and Zonula occludens-1 (ZO-1) against LPS-stimulated damage and inhibiting the ERK-1/2-MAPK pathway [52].

Endothelial glycocalyx is a complex network of proteoglycans, glycoproteins, glycosaminoglycans and plasma proteins located on the surface of endothelial cells. It is an anti-adhesion and anticoagulant layer that maintains vascular integrity. The degradation of endothelial glycocalyx might be one of the important causes of coagulation dysfunction. Markers of glycocalyx degradation, such as Syndecan-1 and HS, are regarded as diagnostic tools in sepsis [53]. Heparin reduces the endothelial glycocalyx loss of the blood–brain barrier in brain injury of status epilepticus [54,55]. 

HPA is an endo-β-D-glucuronidase that degrades the HS side chain at specific in-chain sites. Heparin prevents the loss of endothelial glycocalyx induced by LPS as a competitive antagonist by inhibiting the activity of HPA [56]. In the rat model of sepsis induced by LPS, heparinoids alleviate coagulopathy and glycocalyx loss [57]. Consequently, the levels of glycocalyx components of endothelium including HS and Syndecan-1 recover to normal levels in response to the treatment [58]. Indeed, heparin significantly reduces LPS-induced production of IL-1β, IL-6, E-selectin and ICAM-1, as well as phosphorylation of p38 MAPK and NF-κB translocation, protecting against endothelial-cell-mediated immune response [59]. 

Sulodexide is a heparinoid with high anti-inflammatory potential, mainly composed of fast-mobility heparin and dermatan sulfate. Sulodexide has displayed significant endothelium-protective effects in diabetic nephropathy, ischemia–reperfusion damage, and acute lung injury of the heart, kidney and lung [60]. The drug inhibits the shedding of Syndecan-1, restores the heparinase III-induced suppression of ZO-1, and reduces the vascular permeability [61]. The protective effect is mostly attributed to the endoplasmic reticulum stress reduction via the phosphatidylinositol 3-kinase/Akt signaling pathway [62].

Recently, a heparinoid inhibitor CV122 (-1Me-GlcNS6S-IdoA2s-GlcNS3S6S-IdoA3S-GlcNS6S-) targeting HPA was rationally designed and synthesized based on the structure–activity relationship between the crystal structure of HPA and its substrate. The compound was proven to efficiently alleviate the symptoms of sepsis in three models induced by endotoxin, bacterial and viral infections, respectively. In LPS-induced sepsis, CV122 significantly suppresses the release of early cytokines, protects the glycocalyx structure of vascular endothelial cells, reduces the degree of organ damage, improves vitality and raises the survival rate substantially [63]. 

Apart from the above mechanisms, heparin protects the endothelial barrier damaged by LPS by inhibiting the decomposition of MTs [64]. p38/MAPK signaling pathway stimulation is determined as one of the signaling transduction pathways that accounts for the acetylation of tubulin and stabilizing the MTs in human pulmonary microvascular endothelial cells [65]. However, the detailed mechanisms remain unrevealed.

#### 2.3.2. Disrupt the NET Formation

Neutrophils are the earliest components of the innate immune system recruited to the site of infection. They participate in host defense against pathogens through the release of NETs, the web-like structures composed of chromatin decorated with cytoplasmic and nuclear-derived antimicrobial proteins and peptides, with histones as the predominate components (~70%) [66]. NET release is a potent mediator for the prothrombotic disorder through the recruitment of platelets under arterial and venous shear rates by acting as a scaffold to support platelet aggregation and Glycoprotein IIb/IIIa activation [67]. The formation of NETs with various DAMP components including S100 A8/A9, Cathelicidin LL-37 and myeloperoxidase contributes to the onset and progression of pathologies ranging from autoimmune to inflammation disorders [68].

Extracellular HMGB-1 strongly induces the formation of NETs through the TLR-2/4 associated signaling pathways during ischemic brain and acute lung injury. On the other hand, the NET activation, such as DNA-mediated proteolysis by neutrophil elastase enhances the binding activities of HMGB-1 and promotes macrophage proptosis, thus exacerbating tissue damage [69]. Therefore, targeted NET inhibitors were proposed as a promising strategy for intervention and prophylaxis of thrombo-inflammation. 

As heparin simultaneously targets extracellular histones, HMGB-1 as well as thrombin, the drug therapy has represented an efficient strategy for NET degradation. Moreover, the high charge density allows for heparin to act as a potent competitor for negatively charged DNA during the protease binding [70]. In fact, heparin is capable of destabilizing NET structure. For example, NET induces the pathological changes of corneal epitheliopathy, conjunctival cicatrization, ocular surface inflammation and meibomian gland disease; heparin has served as efficient treatment by dismantling NETs and reducing epithelial, fibroblast, T cell and MG cell changes [71]. Low molecular weight heparins (LMWHs) induce a profound change in the ability of human neutrophils to generate NETs and mobilize the content of the primary granules in response to inflammatory stimuli such as IL-8, phorbol 12-myristate 13-acetate and HMGB-1 [70]. 

#### 2.3.3. The Emerging New Mechanisms 

Recently, the modulation of lipid mentalism with heparin during sepsis has become a research hotspot. Jian Liu’s group discovered that an HS octadecasaccharide promoted the dissociation of ApoA-I from high-density lipoprotein, and stimulated non-toxic ApoA-I/LPS complex formation afterward, which accelerated the clearance of LPS from circulation, and reduced the amount of LPS/HMGB-1 complex [72]. Moreover, whether the effects of heparin are mediated by lipid metabolism (for example, lipid raft as the viral entry route) still needs further investigation [73]. Since heparin improves oxygenation during sepsis treatment, the drug might prohibit disease progression by maintaining energy homeostasis [74].

Yang Ji et al. found that heparin derivatives attenuated bleomycin-mediated apoptosis of Beas2B cells [75]. The study describes a distinct role of heparin in protecting against epithelium injury, although there is a lack of deepening research on the downstream signal pathways. Further research is urgently needed to better unravel the more detailed roles that heparin plays in the pathogenesis, and finally select ideal candidates for sepsis therapy.

### 2.4. The Mechanisms for Heparin Defenses against COVID-19

COVID-19 is associated with a significant increase in pro-coagulant factor levels, including fibrinogen and D-dimers, which are direct reasons for mortality [76]. Heparin is considered as one of the most successful agents in tackling COVID-19-associated thrombotic complications. At the same time, SARS-CoV-2 infection leads to multiple instances of endothelial dysfunction and glycocalyx/barrier disruption [77]. Heparin is considered to confer benefits in moderate–severe COVID-19-infected persons potentially by harnessing heparin-mediated endothelial stabilizing effects, thus alleviating the sharp thrombo-inflammatory response [78]. For instance, the serum Syndecan-1 from COVID-19 patients increased over three-fold compared with healthy subjects. Heparin inhibits the glycocalyx perturbation in cultured human umbilical vein endothelial cells stimulated by the plasma from COVID-19 patients [79].

Moreover, heparin has exerted direct antiviral activity. SARS-CoV-2 anchored ACE2 via Sp on the membrane when entering the respiratory epithelial cell. HS on the cell surface works as a co-receptor, promoting Sp-ACE2 interaction, viral infection and local inflammatory responses [80,81,82]. Heparin and its analogues can be employed as decoy receptors to bind with the Sp protein and inhibit viral binding to HS, thereby inhibiting viral attachment. A structure and function relationship study has revealed that hexasaccharide in the presence of 2S or 6S groups is the minimum size required for the activity [83,84].

## 3. The Prophylaxis and Treatment of Sepsis with Heparin in Clinical Trials

Heparin has shown a broad-spectrum of efficacy on sepsis-associated diseases in clinics. Increasing attention has been paid since the role of heparin was gradually recognized as a COVID-19 treatment, especially its pleiotropic activities to inhibit the inflammatory phase preceding pulmonary microthrombi and impaired pulmonary gas exchanges [84,85,86]. However, the evidence for the beneficial effect of heparin remains contradictory [87]. The effects are influenced by the timing of drug administration, dosing and the route of drug delivery [49,88].

The current literature has proposed that early administration of heparin is associated with better outcomes [89]. Therapeutic doses of heparin in moderately ill patients seem to increase the probability of survival with a reduced need for organ support; in contrast, the primary outcome of days would not be improved without organ support in patients who are critically ill and is probably associated with more bleeding complications. Similarly, Sulodexide administered to COVID-19 patients in the early stages is associated with reduced hospitalization and supplemental oxygen rate, and reduced serum D-dimer levels [90,91]. The analysis has attributed the reasons for this to be that the thrombotic and inflammatory damage may be too advanced to be influenced by the heparin in the case of critical illness because the thrombin was bound with fibrin; whereas heparin controls the network of pro-thrombotic and inflammation before conditions become worse [92]. 

Secondly, the level of dose is another issue that affects the efficacy [93]. Therapeutic anticoagulation with heparin significantly increases organ support-free days among non-critically ill patients compared with the standard prophylactic dose with a two-fold increased risk of major bleeding [55]. Therefore, the intermediate doses of heparin that are higher than a standard prophylactic but lower than the recommended therapeutic dose are desirable [94]. In hospitalized patients, a lower risk of thrombotic complications and death with an intermediate dose was found compared with a prophylaxis dose [95]. However, the optimal dose of heparin therapy remains in debate. One solution is to consider variable doses, i.e., give a standard dose for the patients but increase this to an intermediate dose in the presence of an increased thrombosis risk. This strategy increases the survival of hospitalized COVID-19 patients without increasing the major bleeding risk [96].

The effects are related to the administration route of heparin. Several clinical trials were conducted on patients with acute lung injury who inhaled heparin. The results showed reduced pulmonary damage, microvascular thrombosis, and significantly increased time free of ventilatory support. Also, the nebulized heparin therapy improves the inflammatory conditions [97], and self-reported performance of daily physical activities, but less progression of lung injury and hospitalization in patients with or at risk of acute respiratory distress syndrome [98]. In summary, the inhaled heparin increases clinically relevant activated partial thromboplastin time (peaks were within the normal range) [99], mitigates the damaging effects of COVID-19 infection [100], and is associated with an improvement in oxygenation [101] and WHO MOCS for COVID-19 both in intubated and non-intubated patients [102]. 

In addition, a pilot study with nebulized heparin was performed in Brazil. A post hoc analysis was performed to illustrate the safety and efficacy of heparin. The results have shown a statistically significant difference for the primary improvement of mortality in favor of nebulized heparin added to the standard of care in comparison with standard of care only [103]. In all, nebulized heparin therapy has shown numerous positive effects in clinical studies. This type of administration is superior to intravenous administration because it allows for targeted delivery and boosts local efficacy, reducing the risk of bleeding throughout the body [104].

### 3.1. Comparison between the Different Types of Heparins

Considering the structural diversity of heparin responsible for various activities, the well-defined, heparin-derived structures should be optimized that orchestrate the comprehensive functions during COVID-19 therapy, as well as sepsis induced by other reasons in analogy to the heparin-based pentasaccharide (Arixtra/Fondaparinux) that mediates anticoagulation via antithrombin III [105]. A series of nice work has already been carried out by many excellent groups in pre-clinical research. However, there are few works related to safety and efficacy comparisons from different types of LMWHs and heparin in clinical trials, which might provide clues for further structure and activity relationship investigations. 

A randomized, controlled trial enrolled 126 patients who were hospitalized in the intensive care unit with severe COVID-19 and coagulation, and compared the efficacy of heparin and Enoxaparin. The efficacy of heparin with a therapeutic dose is equaled to Enoxaparin at prophylactic and therapeutic doses in reducing the number of intubations and patient mortality. Heparin is advantageous over Enoxaparin by reducing the risk of intubation and death with a prophylactic dose [106]. 

Meanwhile, in a multicenter propensity-score matched study, the Enoxaparin application was also associated with reduced thrombosis events using logistic regression analysis compared with heparin. The 30-day and in-hospital mortality were similar between the two groups [107]. Although it seems too late to interfere with heparin therapy, LMWHs are associated with a higher cumulative survival rate in a single-center retrospective cohort study [108].

### 3.2. Severe Acute Pancreatitis (SAP) Therapy

Acute pancreatitis is a serious inflammatory condition. Up to one-third of patients develop pancreatic tissue necrosis with a mortality rate of 30%. SAP is usually characterized by a rapid onset of systematic inflammation. For example, acute lung injury is regarded as the earliest organ dysfunction [109]. The main lethal mechanisms are regarded as the pancreatic microcirculation disturbance due to the release of endothelin, a long-acting vasoconstrictor [110]. Both heparin and heparinoid were shown to reduce cytokine release including IL-6 (80%), TNF-α (50%) and HMGB-1 (over 80%), and reduced the morality with a carrulein, caerulein and LPS, as well as a “Two-Hit” of L-arginine-induced mouse models [111]. 

A series of clinical trials were carried out with different forms of heparin and confirmed multifaceted efficacy. In a randomized, single-blind, phase-III control trial with 1386 acute abdomen cases screened, Enoxaparin intervention in the early stage of moderate and severe SAP significantly reduced the chances of disease progression, pancreatic necrosis, local complications, incidences of systemic complications such as ARDS and MODS and mortality rates [112]. The results clearly indicated that the application of LMWHs at an early stage of the disease significantly reduced the chance of disease progression and pancreatic necrosis. 

Meanwhile, LMWHs are safe and compatible with other conventional therapies for SAP treatment [113]. In an earlier multicenter prospective clinical study with 265 SAP patients, LMWHs exhibited synergistic effects with conventional therapies in the acute physiology and chronic health evaluation II scores, complication rates, mortality and mean hospital stays [114]. Another similar clinical trial demonstrated elevated levels of IL-10 in a combinational group with LMWHs, while sB7-H2, TNF-α, sTREM-1 and IL-1 levels were significantly lower [115]. The symptoms and signs improvement rate, the levels of blood and urine amylase, the CT score, and the Acute Physiology and Chronic Health Evaluation II score were improved, and pulmonary embolism occurrence rate, mortality, and mean hospital stay were obviously lower [116]. With these encouraging results in hand, there is a growing need to carry out larger, multicenter clinical trials to evaluate the potential benefit of heparin therapy [117].

It is noteworthy that intensive insulin combined with heparin therapy has displayed higher efficacy on SAP when the disease is induced by hypertriglyceridemia. Hyperglycemia (HTG) following insulin resistance strongly increases the oxidative stress for monocyte and macrophage pathogenic conditions, resulting in SAP aggravation. Insulin combined with LMWHs enhances the lipoprotein lipase activity, an essential enzyme to eliminate circulating triglyceride, and increases the white blood cell count, hemodiastase level, serum albumin level and the arterial partial pressure of oxygen of the patients. Moreover, the therapy significantly shortens the intestinal recovery time and improves pancreatic microcirculation [118]. Consistent with this, several clinical case reports have confirmed that recurrent seasonal severe HTG-induced SAP could be managed with insulin and heparin [119,120].

Currently, the mechanisms of heparin for SAP therapy are attributed to the improvement of microcirculation, catabolism and anabolism, and the promotion of anti-inflammatory reactions. Other underlying mechanisms of heparin and the synergetic effect between insulin and heparin are still to be explored.

## 4. Discussions and Perspectives

Despite a large amount of success with heparin in the prevention and therapy of SIRS and sepsis, the outcomes from clinical trials are not always satisfactory. The uncertainty is created by the unclear mechanisms. In fact, sepsis affords a very complex and variable nature due to the innate immune and hemostatic responses. It is evident that the appropriate timing of therapeutic interventions for certain patients to regulate sepsis in specific disease states will ultimately be critical for optimized treatment based on a deepening understanding of the molecular mechanisms. Although several new theories are being continually discovered, the determined role of heparin still remains unknown. 

Secondly, great efforts from academic institutions have been made to explore the underlying anti-inflammatory mechanisms of heparin; however, only a few of them were demonstrated in clinical trials (Table 1). In fact, the link between preclinical studies and clinical trials for severe acute inflammation therapy was initiated during the COVID-19 pandemic. For example, accumulating clinical evidence has implicated the imbalance between NET formation and disassembly plays a central role in the pathophysiology of inflammation, coagulopathy, organ damage and immuno-thrombosis that characterize severe cases of COVID-19 [121]. Both heparin and LWMHs neutralize extracellular cytotoxic histones, accelerate DNaseI-mediated degradation of NET-mediated clots and prevent NET aggregation in COVID-19 patients [121]. The therapeutic value of heparin was also demonstrated by the improvement of lung oxygenation, prevention of ARDs and reduction of mortality [122,123]. In addition, Sulodexide was shown to protect endothelial glycocalyx and restore endothelium integrity after lesions in preclinical studies. As such, Sulodexide may prevent the development of endothelial lesions in patients with mild COVID-19 by 40% and hence inhibit the progression of inflammation and coagulopathy [124]. 

However, the results in clinical trials are only limited to primary efficacy and coagulation parameter investigations in sepsis response to other infections. The biochemical biomarkers that are related to new molecular mechanism discoveries are currently rarely validated in clinical trials.

The relationship between structure and function is still a hot issue during drug development to reduce the risk of side effects and increase efficacy. Particularly, the reduction of heparin-induced thrombocytopenia (HIT), a life-threatening symptom that promotes NET formation, should be taken seriously when the drug is utilized in clinics [125,126]. The occurrences of HIT would increase as the balance of formation vs. disassembly of NETs is very weak, especially in the context of sepsis which is prone to NET formation. Therefore, a homogeneous structure design to completely avoid the HIT effect would be necessary. Fortunately, the synthesis of structurally new heparin and heparinoid no longer perplexs the research [3,127]. With progression in their underlying mechanisms, heparin and heparinoid would be developed as a promising drug for alleviating SIRS and sepsis.

**Table 1:** The clinical research of heparin and the associated mechanisms related to acute severe inflammation and SIRS.

Disease	Mechanism of the Effects	Clinical Progress	Notes
Sepsis/acute lung injury	Improve the dysfunction of endothelial barrier. Decrease p-MYPT1 expression in lung tissue. Inhibit phosphorylation of p38, alveolar coagulation and early inflammatory responses. Inhibit the decomposition of microtubules and the expression of guanine nucleotide exchange factor, stabilize the microtubules, and improve F-actin remodeling in endothelial cells. Disrupt NET formation.	✓ Nebusolized heparin targeting fibrin limits lung damage and accelerates physical function recovery in patients with ARDS or at risk for ARDS. The heparin group had fewer cases of ARDS develop by day 5 among the at-risk patients (15 vs. 30%), less deterioration of the MLIS by day 5 (difference −0.14, *p* = 0.0215), and more day 60 survivors at home (8% vs. 73%, *p* = 0.0165) [98]. ✓ Heparin inhalation reduces lung damage in burn patients [128]. ✓ The retrospective observational study demonstrates that adult sepsis patients administrated with heparin had significant survival benefits with moderate coagulopathy and sepsis-induced coagulopathy scores (adjusted OR: 0.452, 0.625; 95% CI: 0.265–0.751, 0.410–0.940, respectively) [129].	
		⌧ Patients at the emergency room with signs indicative of sepsis were administered heparin. The overall 28-day mortality was 16% in the placebo group and 14% in the heparin group (*p* = 0.652). Subgroup analyses did not show any statistically significant reduction in 28-day mortality with heparin [19].	The experiment design does not consider the proper time and patients for heparin therapy.
COVID-19	(1) Anticoagulant effects. (2) Prevent inflammatory cell infiltration by interacting with chemokines such as chemokine ligand C-X-C motif chemokine ligand 12 and binding to P/L-selectin to inhibit recruitment and adhesion of neutrophils and leukocytes. (3) Protecting the integrity of glycocalyx of endothelial cells and inhibition of NETs via targeting histone, HMGB-1 and HPA etc. (4) Being employed as decoy receptors to bind to the Sp protein and inhibit viral binding to HS.	✓ Patients with laboratory-confirmed severe acute respiratory syndrome are administrated with heparin or LMWHs. The results: death rate for patients receiving heparin was reduced by over 40% (7.4 vs. 14.0 per 1000 people) [18]. ✓ In non-critically ill patients, an initial use strategy of therapeutic dose anticoagulation with heparin increases the probability of survival to hospital discharge (80.2 vs. 76.4%) with reduced use of organ support [21]. ✓ In non-critically ill patients, therapeutic-dose heparin is associated with a 97.7% probability of superiority to reduce the composite of stage 3 acute kidney injury or death (3.1 vs. 4.6%) [130]. ✓ Thromboprophylactic Enoxiparin has no difference in the composite of all-cause mortality and hospitalization at 21 days between the Enoxaparin (12 of 105 patients, 11%) and the control group (12 of 114 patients, 11%, *p* = 0.83) [88]. ✓ Early (<48 h) administration of heparin is associated with reduced utilization of invasive mechanical ventilation (OR 0.23, *p* ≤ 0.01) and non-IMV (OR 0.49, *p* = 0.03) and reduced ICU (MD-1.64, SE 0.58, *p* ≤ 0.01 and hospital length of stay (MD-4.15, SE 0.93, *p* ≤ 0.01) [89]. ✓ Inhaled LMWH significantly improves hypoxemia [99,101]. ✓ The mortality is numerically lower for nebulized heparin (6 out of 38 patients, 15.8%) vs. control group (10 out of 37 patients, 27.0%), but not statistically significant [103]. ✓ Patients treated with LMWHs at a prophylactic dose but not at a therapeutic dose as compared to patients treated with heparin show an increased risk of intubation (*p* = 0.026, OR = 3.33, 95% CI = 1.15–9.59), and an increased risk of death (*p* = 0.046, OR = 3.01, 95% CI: 1.02–8.90). ✓ Patients who received heparin compared to Enoxaparin have higher all thrombosis events at crude analysis (18.3 vs. 4.6%, *p* = 0.02) [107]. ✓ Sulfoxide diminishes the score of circulating endothelial cells by 40% in mild viral infection patients. In the drug group, it significantly decreases from 5.7 ± 1.7 to 2.4 ± 0.9 (*p* = 0.03) [124]. ✓ When Sulodexide is provided within 3 days of clinical onset, the hospitalization rate is reduced from 29.4 to 17.7%, the rate for supplemental oxygen is reduced (30 vs. 42%). High D-dimer level rate is reduced (22 vs. 47%, *p* < 0.01) [90]. ✓ Sulodexide alleviates chest pain (83.7 vs. 43.6%, *p* < 10^−3^), palpitations (85.2 vs. 52.9%, *p* = 0.009), and endothelial function [median delta-EQI 0.66 (0.6) vs. 0.18 (0.3), *p* < 10^−3^] [131].	
		⌧ In critically ill patients with COVID-19, an initial strategy of therapeutic-dose anticoagulation with heparin does not result in benefits, the survival rate was 62.7 vs. 64.5% [22].	Heparin might intervene with the inflammatory reaction in the early stage of sepsis that initiates the coagulation cascade.
Acute kidney injury	Reduces H4 (histone) levels and the release of pro-inflammatory cytokine in HK-2 cells (human renal tubular epithelial cells).		
Acute severe pancreatitis	(1) Anticoagulant effect. (2) The active secretion of HMGB-1 is inhibited by blocking the ectopic location in the macrophage nucleus. (3) Improve the microcirculation, catabolism and anabolism, and the promotion of anti-inflammatory reactions. (4) The insulin combined with LMWH enhanced the lipoprotein lipase activity, an essential enzyme to eliminate circulating TG.	✓ LMWH administration significantly improved the levels of blood and urine amylase, the CT score (*p* < 0.05–0.01), and reduced pulmonary embolism occurrence rate, mortality, and mean hospital stay (*p* < 0.05–0.01) [116]. ✓ LMWH results in reduced index progressed in (8.6 vs. 31.4%), and necrosis (5.71 vs. 25.71%) [112]. ✓ LWMHs combined with ulinastatin showed a shorter time to return to normal (*p* < 0.05). The levels of IL-10 in LWMH group were higher, while sB7-H2, TNF-α, sTREM-1 and IL-1 levels were lower than those (*p* < 0.05) [115].	
Acute liver injury	By targeting the HMGB-1 /RAGE axis, the chemotaxis of neutrophils is inhibited, the anti-inflammatory effect on the damaged liver is enhanced and the efficacy is significantly improved.	✓ Heparin reduced the risk of post hepatectomy liver failure (odds ratio: 0.518; 95% confidence interval: 0.295–0.910, *p* = 0.022), and was associated with improved short-term post operative outcomes such as reduced ICU stay durations, diminished requirements for respiratory support, and lower incidences of hypoxemia and ICU mortality [132].
